# Convergence and Divergence: Signal Perception and Transduction Mechanisms of Cold Stress in *Arabidopsis* and Rice

**DOI:** 10.3390/plants10091864

**Published:** 2021-09-09

**Authors:** Xiaoshuang Wei, Shuang Liu, Cheng Sun, Guosheng Xie, Lingqiang Wang

**Affiliations:** 1 State Key Laboratory for Conservation and Utilization of Subtropical Agro-Bioresources, College of Agriculture, Guangxi University, Nanning 530004, China; 1917301040@st.gxu.edu.cn; 2 MOA Key Laboratory of Crop Ecophysiology and Farming System in the Middle Reaches of the Yangtze River, College of Plant Science and Technology, Huazhong Agricultural University, Wuhan 430070, China; 2018301110013@webmail.hzau.edu.cn (S.L.); 13707255053@webmail.hzau.edu.cn (C.S.)

**Keywords:** cold stress, signal perception, signal transduction pathways, *Arabidopsis*, rice

## Abstract

Cold stress, including freezing stress and chilling stress, is one of the major environmental factors that limit the growth and productivity of plants. As a temperate dicot model plant species, *Arabidopsis* develops a capability to freezing tolerance through cold acclimation. The past decades have witnessed a deep understanding of mechanisms underlying cold stress signal perception, transduction, and freezing tolerance in Arabidopsis. In contrast, a monocot cereal model plant species derived from tropical and subtropical origins, rice, is very sensitive to chilling stress and has evolved a different mechanism for chilling stress signaling and response. In this review, the authors summarized the recent progress in our understanding of cold stress response mechanisms, highlighted the convergent and divergent mechanisms between *Arabidopsis* and rice plasma membrane cold stress perceptions, calcium signaling, phospholipid signaling, MAPK cascade signaling, ROS signaling, and ICE-CBF regulatory network, as well as light-regulated signal transduction system. Genetic engineering approaches of developing freezing tolerant *Arabidopsis* and chilling tolerant rice were also reviewed. Finally, the future perspective of cold stress signaling and tolerance in rice was proposed.

## 1. Introduction

Cold stress limits the geographical distribution, growth habits, and productivity of plants [1]. There are two types of cold stress, including chilling stress (0 to 15 °C) and freezing stress (below 0 °C). Many temperate plants, such as *Arabidopsis*, rapeseed, wheat, and rye, have acquired a complex network that senses and responds to freezing stress. Molecular mechanisms and regulation networks of plasma membrane cold perception, signaling and freezing stress tolerance of these plants have been well-reviewed [2,3,4,5,6,7,8,9]. On the other hand, rice (*Oryza sativa* L.), one of the most important cereal crops, is susceptible to chilling stress, especially in high elevation and high latitude temperate zones [10]. Many novel regulatory genes have been identified in chilling stress perception and signaling in rice in the past decade [11,12,13]. This allows us to compare and distinguish the similarities and differences of cold stress sensing and signaling mechanisms between *Arabidopsis* and rice [8,9].

Plants respond to the decreased temperatures through sophisticated processes at different levels. Initially, plant cells perceive cold stress signals through the plasma membrane (PM) rigidification, PM-bound G-protein associated receptors, or cold sensors, such as Ca^2+^ influx channels, two-component histidine kinases, protein and nucleic acid conformations, or metabolite concentrations [6,14]. Then, the second messengers, including calcium ion, reactive oxygen species (ROS), and inositol phosphates, are generated. These second messengers further modulate the intracellular calcium (Ca^2+^) level. Perturbation in cytosolic Ca^2+^ level is sensed by calcium-binding protein (Ca^2+^ sensors), which interact with their target proteins, to transduce calcium signal in the cell. These proteins orchestrate cold stress signal transduction, activate protein phosphorylation cascades, and adjust the expression of transcription factors and cold regulated (*COR*) genes in plants [6].

This review summarized the recent progress in (i) cold stress perceptions, including PM-associated receptor-like kinases (RLK) and cold sensors; (ii) cold stress signaling mechanisms, including calcium signaling, phospholipids signaling, MAPK (mitogen-activated protein kinase) cascade signaling, and ROS signaling; (iii) ICE (inducer of CBF expression)-CBF (C-repeat binding factor) transcriptional regulatory network; (iv) the light-regulated signal transduction system in cold stress tolerance. We highlighted the similarities and differences in cold-induced responses contributing to freezing tolerant *Arabidopsis* and chilling tolerant rice. Finally, we evaluated the genetic engineering for improving cold stress tolerance in plants and explored future research of cold stress signaling and tolerance mechanism in rice.

## 2. Cold Stress Perceptions at Plasma Membrane (PM) in *Arabidopsis* and Rice

**Figure 1:**
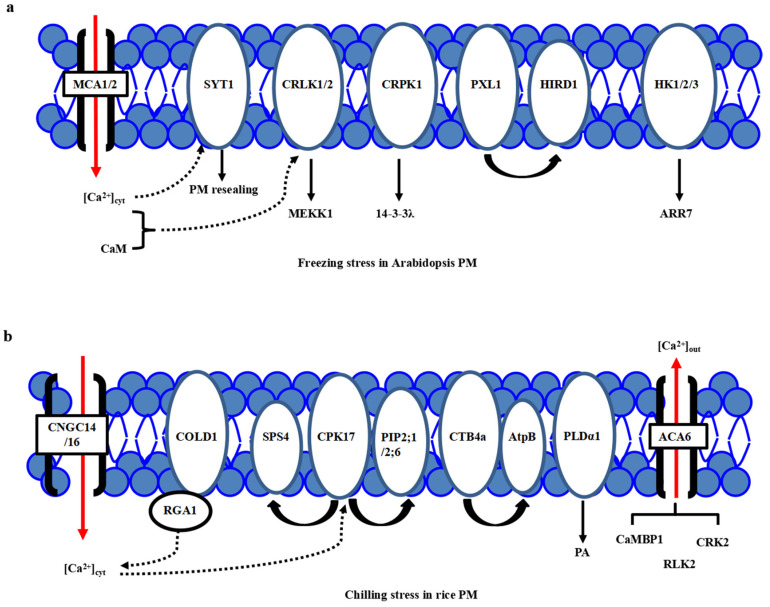
Plasma membrane-localized proteins perceive the cold stress signals in *Arabidopsis* and rice. (**a**) In *Arabidopsis*, freezing stress initiates the PM rigidification, PM-associated calcium channels MCA1/2, calcium sensor SYT1 and kinases including CRLK1, AtHK1/2/3, and CRPK1, as well as PM-localized PXL1, participate in primary cold stress sensing and perception. (**b**) In rice, chilling stress initiates the PM rigidification, many PM-associated proteins, including calcium channels ACA6, CNGC14/16, phospholipidase PLDα1, aquaporin proteins PIP2;1/PIP2;6, G-protein-associated cold sensor COLD1 and kinases GT4a and CPK17, participate in primary cold stress sensing and perception. However, a specific calcium channel for calcium influx is still not known.

One of the major consequences of the temperature downshift is a decrease in membrane fluidity affecting membrane-associated cellular functions, and the PM is proposed as a primary sensor of low-temperature stress [15,16,17]. The feature of primary perception of temperature in plants has been proposed [18]. Different microdomains with lipid raft formation and composition, including sphingolipids in the PM, are responsible for sensing the particular temperature ranges [8,15]. Many putative calcium channels, PM-bound G-protein associated receptors, plasma membrane-localized receptor-like kinases (RLKs) have been identified as cold sensors in plants. Calcium channels responsible for Ca^2+^ influx have been considered a major sensor class for low temperature [19,20,21]. Through the membrane rigidification-activated mechano-sensitive or ligand-activated Ca^2+^ channels, cold stress induces a transient Ca^2+^ influx into the cytosol (Figure 1a). Two *Arabidopsis* calcium-permeable mechano-sensitive channels, AtMCA1 and AtMCA2, are involved in a cold-induced increase in [Ca^2+^]_cyt_ and cold stress tolerance [22]. A cold sensor OsCOLD1 is the novel PM and endoplasmic reticulum (ER)-located protein, which interacted with α subunit 1 of the G protein (RGA1), enhancing the calcium transients in the cytosol in cold signal transduction in rice [23]. Two cyclic nucleotide-gated channels, OsCNGC14 and OsCNGC16, mediate the calcium signaling and promote chilling tolerance in rice seedlings. Their homologous proteins AtCNGC2 and AtCNGC4 in *Arabidopsis* promote chilling growth and freezing tolerance [24]. OsCNGC9 positively regulates chilling tolerance by mediating cytoplasmic calcium signaling in rice [25] (Figure 1b). Therefore, calcium channels play a central role in cold stress sensing in *Arabidopsis* and rice.

On the other hand, a new calcium sensor synaptotagmin without the EF-hand motif, AtSYT1, localized to the PM and ER, participates in the exocytosis process in the calcium-dependent pathway under freezing stress in *Arabidopsis* [26] (Figure 1a). In rice, thirteen SYT homologous N-terminal-TM-C2 domain proteins (OsNTMC2) have been annotated [27]. However, the function of OsNTMC2 in vesicle trafficking and PM repair in cold stress response awaits further investigations.

Many receptor-like protein kinases, such as two-component histidine kinases, RLKs, and G-protein associated kinases, have played pivotal roles in cold stress sensing in *Arabidopsis* and rice. Two-component signaling systems, AHK2/3, AHP2/3/5, and ARR7, mediate the cold stress signaling through inhibiting ABA signaling [28,29]. Besides, AtCRLK1 binds to calcium and calmodulin (CaM), interacts with phosphorylates AtMEKK1 in freezing signaling and tolerance [30]. Moreover, AtCRPK1 phosphorylates 14-3-3λ which shuttles from the cytosol to the nucleus, then interacts with and destabilizes the CBFs in freezing stress tolerance [31]. In addition, AtPXL1 interacts with and phosphorylates histidine-rich dehydrin1 (AtHIRD1) and a light-harvesting protein complex I (AtLHCA1) to positively regulate cold and heat stress tolerances during the germination stage [32].

In rice, there are several identified PM-localized kinases involved in cold stress perception. OsACA6, a PM Ca^2+^-ATPase, interacts with CaM-binding protein OsCaMBP1, calcium-dependent protein kinase (CDPK)-related kinase OsCRK2, and receptor-like kinase (RLK) OsRLK2 [33]. PM-localized OsCPK17 interacts with and phosphorylates the sucrose-phosphate synthase OsSPS4 and aquaporin OsPIP2;1/OsPIP2;6, can enhance the cold stress tolerance in rice [34](Figure 1b). CTB4a, a conserved leucine-rich repeat receptor-like kinase, interacts with a beta subunit of adenosine triphosphate (ATP) synthase AtpB and improves the yield under cold stress [35]. Interestingly, the protein level of phospholipase Dα1 (OsPLDα1) increases at one minute after cold treatment. It activates OsMPK6 and OsSIZ1, followed by the regulations of OsDREB1s expression in cold signaling [36] (Figure 1b). Therefore, there is much convergence of primary PM-located protein kinases in cold stress perceptions between *Arabidopsis* and rice.

## 3. Cold Stress Signal Transduction Mechanisms in *Arabidopsis* and Rice

**Figure 2:**
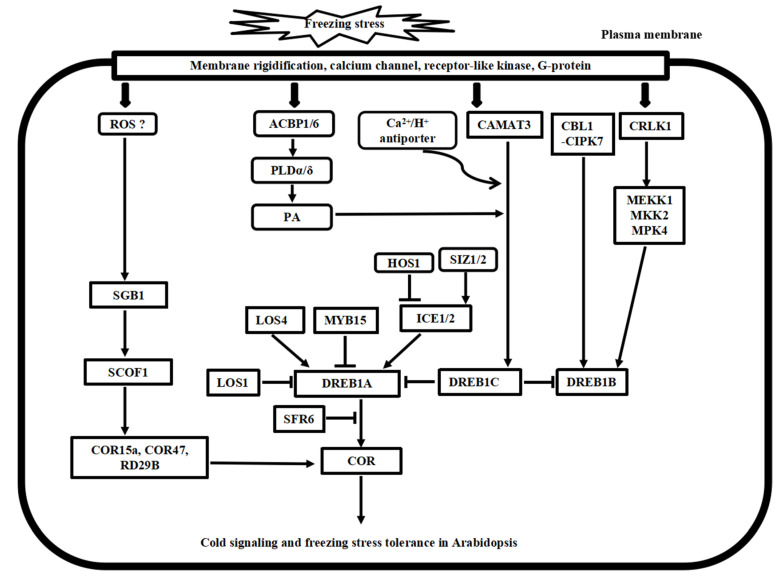
Putative model of cold stress signaling networks toward freezing stress tolerance in *Arabidopsis*. The cold-induced calcium signature in the cytosol is recognized by the calcium sensor proteins, including CaM, CDPK, CBL1/CIPK7, and CAMAT3, as well as the bZIP transcription factor SGB1 pathway. In addition, CRLK1-MEK1-MKK1/2-MPK4/6 cascade, ROS signaling, and phospholipid signaling work together to regulate cold stress signaling, and many ICE1-DREB transcription activators and repressors have been identified to regulate the *COR* gene expressions, finally leading to freezing tolerance in *Arabidopsis*.

**Figure 3:**
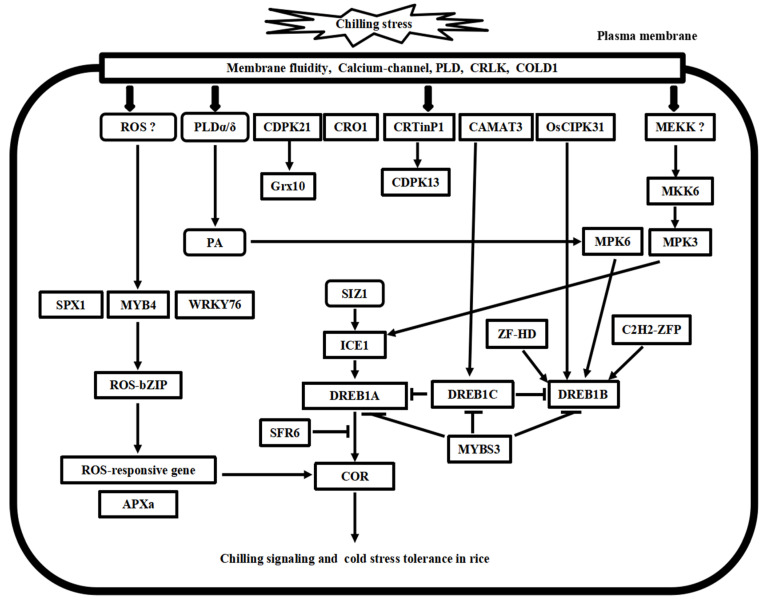
Putative model of chilling stress signaling networks toward cold stress tolerance in rice. There are at least four chilling stress signaling pathways in rice. MYB4-ROS-bZIP cascade is involved in the ROS signaling process. MKK6-MP3 cascade and phospholipid signaling work with calcium signaling concomitantly in chilling stress signaling in rice. ICE1-DREB transcriptional regulatory cascade is conserved in the *Arabidopsis* and rice. Furthermore, these pathways’ upstream and downstream signal transducer proteins play cooperative and regulatory roles in cold stress tolerance in rice.

Following the cold stress perceptions, cold stress signal transduction events occur in the cytosol and nucleus of plant cells. The second messengers, such as Ca^2+^ and reactive oxygen species (ROS), transmit the external cold signals to intracellular signaling systems. Progress has been made in calcium signaling, phospholipid signaling, MAPK cascade signaling, and ROS signaling in the past decades. Here, we compare the recent advances in signal transduction pathways of freezing stress in *Arabidopsis* and chilling stress in rice (Figure 2 and Figure 3), highlighting the divergence and convergence in cold stress in both plant species.

### 3.1. Calcium Signaling

Calcium influx into the cytosol is an early event in cold stress [6,17]. This transient elevation in calcium concentration is also called intracellular calcium signature. Calcium influx is primarily sensed by the calcium sensor proteins, containing the helix-loop-helix domain with the EF-hand motif. In plants, calcium sensors include four major classes: CaM/CaM-like protein (CML), calcium-dependent protein kinase (CDPK or CPK), calcineurin B-like (CBL) protein, and CBL-interacting protein kinase (CIPK). In addition, a small annexin family has been identified as a calcium sensor to cold stress response in *Arabidopsis*.

In *Arabidopsis*, overexpression of *AtCaM3* hinders the cold induction of *RD29A* and *KIN1*, and the AtCaM4 negatively regulates freezing tolerance by interacting with a CaM-binding protein PATL1 [37]. AtCBL1 interacts with AtCIPK7 and binds to the DREB core element of *COR* promoters to negatively regulate freezing tolerance [38]. CaM-binding transcription activator protein CAMTA3 binds to the conserved CG-1 element in the *CBF2* promoter, regulating *CBF2* expression in cold stress signaling [39]. A vacuolar Ca^2+^/H^+^ antiporter AtCAX1 enhances the *DREB1* transcription in cold acclimation response [40]. Recently, an AtOST1-AtANN1cascade was found to regulate calcium signaling in the CBF1-dependent manner to enhance freezing tolerance in *Arabidopsis* [41]. This evidence demonstrated the negative and positive regulations of calcium sensors to freezing tolerance in *Arabidopsis*.

In rice, a CaM-like protein OsCML16 and its six putative targets have been identified to be involved in cold stress response in rice [42]. However, there is no report about the role of OsCaMs in cold stress signaling. *OsCDPK7* enhances cold stress tolerance by the increased accumulation of a putative target gene *rab16A* [43,44]. OsCDPK13 enhances cold stress tolerance by activating a ubiquitin-like nuclear protein OsCRTintP1, calreticulin interacting protein 1 [45,46]. OsCPK24 interacts with and phosphorylates OsGrx10, a glutathione-dependent thioltransferase, in cold stress response [47]. Overexpression of *OsCIPK3,* a CBL-interacting protein kinase, improves cold stress tolerance [48]. It is worth mentioning that OsCIPK31 is strongly induced by cold and salt stress and interacts with AtCBL3, suggesting the convergence of CBL/CIPK pathways in cold stress signaling *Arabidopsis* and rice [49]. As described above, the CaM-associated signaling pathways in cold stress signaling wait for further confirmations in *Arabidopsis* and rice.

### 3.2. Phospholipid Signaling

An increasing number of studies have shown that the metabolism of the membrane lipids plays an important role in the temperature stress response in plants. In *Arabidopsis,* in a few seconds after cold exposure, diacylglycerol kinase (DGK) is activated to converse diacylglycerol (DAG) into phosphatidic acid (PA), followed by a change in membrane fluidity [50]. Overexpression of a PM-bound phospholipase gene *PLDδ* enhances freezing tolerance in rice seedlings [51]. Suppressed expression of *PLDα1* results in a significant increase in freezing tolerance [52]. Acyl-coenzyme A: diacylglycerol acyltransferase DGAT1 enhances freezing tolerance via *CBF2* regulon and NADPH oxidase RbohD (respiratory burst oxidase homolog D)-dependent H_2_O_2_ production in *Arabidopsis* [53]. The acyl-coenzyme A-binding protein (ACBP) family has six members (AtACBP1-6) in *Arabidopsis*. Overexpression of *AtACBP6* enhances freezing tolerance by activating PLDδ to decrease phosphatidylcholine (PC) levels and accumulate PA [54]. Overexpression of *AtACBP1* increases freezing sensitivity via the expression of *PLDα1* and *PLDδ* and maintains a membrane-associated PA pool [55]. Further, a temperature-induced lipid pathway has been demonstrated. The FAD2, FAD5 and ACT1 have been identified as the key enzymes in influencing fatty acid flux between the eukaryotic and prokaryotic pathways cold stress response in *Arabidopsis* [56].

In plants, glycerol-3-phosphate acyltransferase (GPAT) of chloroplasts is a key enzyme to catalyze transferring the acyl group of acyl-(acyl-carrier-protein) (ACP) into the sn-1 position of glycerol 3-phosphate in the first step of glycerolipid biosynthesis in chloroplasts. Ectopic overexpressing of *AtGPAT* in rice largely induces the unsaturation of fatty acids and chilling tolerance of photosynthesis under low temperature [57]. In rice, the ω-3 fatty acid (FA) desaturase (FAD8) mutant does not acclimate to cold stress [58]. *OsPLDα1* increases the levels of PA that bind to OsMPK6 in cold signaling and tolerance [36]. Interestingly, comparative glycerolipidomics analysis of freezing stress (−6 °C and −12 °C) in *Arabidopsis* and chilling stress (4 °C and 10 °C) in rice has illustrated that *Arabidopsis* has a higher double bond index (DBI) and lower average acyl chain length (ACL) than rice under cold stress condition [59]. Accordingly, glycerolipid metabolism and signaling show great potentials in applying cold stress tolerance engineering in *Arabidopsis* and rice.

### 3.3. MAPK Cascade Signaling

In plants, the MAPK cascade consists of three sequentially phosphorylating and activating components, a MAP kinase kinase kinase (MEKK/MAPKKK), a MAP kinase kinase (MEK/MAPKK), and a MAP kinase (MPK/MAPK). MAPKs phosphorylate various downstream substrates, including transcription factors, protein kinases, phospholipases, and cytoskeleton-associated proteins, finally leading to the activation of specific gene expressions under stress conditions [60].

In *Arabidopsis*, MAPKKK protein AtANP1 initiates a phosphorylation cascade with AtMPK3 under cold stress [61]. The complete cascade AtCRLK1-AtMEKK1-AtMKK2-AtMPK4/6 has been established to positively regulate freezing tolerance [62]. Recently, AtMPK6 is found to phosphorylate AtMYB15 to reduce the binding affinity of AtCBF3 and freezing tolerance [63]. AtMPK3 and AtMPK6 phosphorylate AtICE1 to promote its degradation, thereby negatively regulate freezing tolerance [64]. AtMPK6 phosphorylates AtMYB5 to positively regulate freezing tolerance [63]. However, the AtMEKK1-AtMKK1/2-AtMPK4 cascade promotes freezing tolerance by antagonizing the AtMPK3/6 pathway [65]. These results indicate that AtMPK3, AtMPK4, and AtMPK6 proteins cooperatively regulate freezing tolerance in *Arabidopsis*.

In rice, there is not identified complete MAPK pathway involved in cold stress signaling until now. Our previous study established that the OsMKK6-OsMPK3 cascade modulates chilling signaling and tolerance in rice [66]. OsMPK3 phosphorylates and stabilizes OsICE1, which directly transactivates the expression of *OsTPP1*, thereby positively regulating chilling tolerance [67]. Moreover, PA binds to OsMPK6 and mediates chilling stress signaling and tolerance [36]. Therefore, these results have demonstrated a divergence in MAPK signaling pathways and regulation network in cold stress response in *Arabidopsis* and rice.

### 3.4. ROS Signaling

Under cold stress, excess ROS is produced and brings about oxidative damage and cold stress response in plant cells. In *Arabidopsis*, AtMEKK1-AtMKK2-AtMPK4/AtMPK6 cascade regulates the ROS-scavenging enzymes to maintain redox homeostasis under cold stress [62]. Overexpression of a ROS-regulated C_2_H_2_ zinc finger transcription factor *AtZAT12* decreases the expressions of *AtCBF1/2/3* genes under cold stress [68]. In addition, AtHAP5A, a heme-associated protein, positively modulates the freezing resistance by binding *AtXTH21* and inhibits ROS accumulation under freezing stress [69]. Stromal and thylakoid-bound ascorbate peroxidases sAPX and tAPX trigger *COR15A*, *PAL1,* and *CHS* expressions under cold stress [70]. AtTrx-h2 regulates the expressions of *COR* genes under freezing stress [71].

H_2_O_2_ levels are increased within 1.5 h of 10 °C stress in rice seedlings [72]. A subset of 121 early-response genes was upregulated during the initial 24 h of 10 °C stress [73]; Among them, four are transcription factor genes, including ROS-bZIP1 and asl/ocs-like element-containing genes. A hypothetical model of ROS-mediated regulon (ROS-bZIP-as1/ocs) is assembled independent of CBF/DREB- or ABA-mediated regulons in cold stress response [72,73]. Comparative metabolomics analysis of *indica* (*9311*) and *japonica* (*Nipponbare*) varieties revealed a ROS-dominated dynamic model involved in chilling stress adaptation and tolerance in rice [74]. Overexpression of *OsZFP245* enhances cold stress tolerance by regulating proline levels and ROS-scavenging activities in rice seedlings [75]. Overexpressing *OsAPX1* prevents the over-accumulation of H_2_O_2_ and reduces lipid peroxidation in the spikelet tissues at the booting stage of rice [76]. Natural variation reveals that OsSAP16 controls low-temperature germination in rice [77]. Therefore, there exist specific and different pathways of ROS-mediated cold signaling in rice.

## 4. ICE-CBF Transcriptional Cascade

ICE and CBF homologs are highly conserved in *Arabidopsis* and rice. In *Arabidopsis*, ICE-CBF transcriptional cascade has been established as the main regulatory response toolkit to cold signaling and freezing tolerance [78]. There are several identified positive and negative regulators, including HK2/3, CAMTA3, OST1, HOS1, and SIZ1/2 for ICE1, ICE1 for CBF3, MYB15 for CBFs, and SRF6 for *COR* [79,80,81]. ICE1 binds toe *CBF3* promoter and induces the *CBF3* expression in cold stress signaling [82]. Overexpression of *ICE2* induces the expression of *CBF1* and enhances freezing tolerance [83]. A RING-finger ubiquitin E3 ligase, HOS1, interacts with ICE1 and targets for polyubiquitylation and proteolysis of ICE1 after 12 h of cold stress [79]. SIZ1 catalyzes sumoylation of ICE1 during cold acclimation and thus reduces the ICE1 polyubiquitylation [80]. OsSFR6 enhances freezing tolerance in Arabidopsis [84]. However, there are no such reports of cold signaling in rice.

Furthermore, OST1 phosphorylates and stabilizes ICE1 to enhance freezing tolerance [85]. ICE1 interacts with and negatively regulates the expression of *MYB15* during cold acclimation [81]. *EIN3,* an ethylene-insensitive 3 gene, negatively regulates the expressions of *CBFs* and type-A *ARRs* [86]. Two repressors of jasmonate acid (JA) signaling, JAZ1, and JAZ4, interact with and suppress *ICE1* and *ICE2* transcription activity, finally repressing the *CBF3* expression and freezing tolerance [87]. ZAT12 regulon inhibits the CBF cold-response regulatory pathway [68]. CAMTA3, a CaM-binding transcription factor, binds to the CG-1 element in the promoter of *ZAT12* under cold stress [28].

In rice, transcript levels of *OsICE1* and *OsICE2* remain constant during cold stress, indicating that they are posttranslationally modified under cold stress [88]. Ectopic overexpression of *OsICE1* and *OsICE2* in *Arabidopsis* imparts freezing tolerance by inducing *CBF* expressions [89]. *OsDREB1A* and *OsDREB1B* transcripts are induced within 40 min after cold stress. Overexpressing *OsDREB1A* increases chilling stress tolerance in rice [90]. Ectopic overexpression of *OsDREB1A* and *OsDREB1B* in *Arabidopsis* enhances the expressions of stress-inducible genes *rd29a*, *cor15a,* and *rd17* [91]. In particular, *OsMYBS3* represses the transcriptional level of the *OsDREB1*-dependent cold response pathway in rice [92]. *OsROC1* enhances the chilling tolerance by activating *OsDREB1* in rice [93]. These results have revealed the convergence in the ICE-CBF cascade and divergence in the regulation of the ICE-CBF cascade in cold stress signaling in both species. As expected, new kinases, phosphatases, and transcriptional regulatory factors upstream and downstream of DREBs will integrate the convergent and divergent networks of cold stress signaling in *Arabidopsis* and rice.

## 5. Light Modulates the Cold Stress Tolerance

Cross talk between light signaling and cold signaling has been elucidated in the model plant *Arabidopsis* and tomato and cereals species [13]. For example, in *Arabidopsis*, there are five phytochromes isoforms phyA-E. By using *Arabidopsis* phys mutants, Franklin and Whitelam confirmed that phyB and D prevent the realization of FR-induced cold tolerance at 16 °C [94,95]. Interestingly, phytochrome A and B function antagonistically to regulate cold stress via abscisic acid (ABA)-dependent jasmonate signaling in tomatoes [96]. Similar results have been obtained in wheat and barley [97]. In barley, the light-regulated signal transduction system was reported to connect the circadian clock, phospholipid signaling pathway, calcium signaling elements, and the downstream *HvCBF3/4* genes expressions under cold stress [98].

In rice, phyB negatively regulates chilling tolerance. The phytochrome B-deficient mutant (*phyB*) shows the more stabilized chloroplast structure and higher unsaturated fatty acid (USFA) content in membrane lipids, thereby alleviating the chilling-induced photoinhibition. Moreover, expressions of genes associated with USFA syntheses such as *OsFAD7* and *OsFAD8* are higher in the phyB mutant than in the wild type, suggesting that OsFAD7 and OsFAD8 are the downstream genes of phyB [99]. On the other hand, phyB negatively regulates the OsDREB1 expression through interacting with OsPIL16 to reduce the membrane integrity under cold stress [100]. Therefore, phyB probably senses the red light signals and transduces the low-temperature signal together with a series of downstream genes, at least in part through changes in the composition and stability of cell membranes under cold stress. Nevertheless, there are many gaps to bridge between red light signal reception and cold stress response in plants.

## 6. Downstream Response Pathways in the *Arabidopsis* Freezing Stress and Rice Chilling Stress

The development of genetically engineered plants by the repression and overexpression of selected genes seems to be a viable option to hasten the breeding of cold stress tolerance-improved crop plants. Genetic engineering of cold stress tolerance was reviewed in crop and horticultural plants in 2011 [101]. The recent progress in freezing tolerant transgenic *Arabidopsis* and chilling tolerant transgenic rice has been updated (Table 1 and Table 2). In *Arabidopsis*, engineering of freezing stress tolerance have been obtained through protein kinases and their associated proteins such as AtLCBK1 [102], AtBZR1 [103], AtCRF4 [104], ARR22 [105], and AtTCF1 [106]. Many transcription factors, such as bZIP, ZFP, WRKY, MYB, NAC, GT, DEAR, AP2, AREB, and ERF, have been applied to improve freezing stress tolerance. Furthermore, AtACBP6 [107] and AtLCBK1 [102] have been identified as positive regulators of freezing tolerance. Based on the four signaling pathways above, these engineered genes function downstream of phospholipid signaling, ROS signaling, and CBF regulon under freezing stress response (Table 1). Thus, these regulatory and functional proteins can adjust the balance of dehydration, detoxification, and metabolism under freezing stress in *Arabidopsis,* increasing the widespread distribution and adaptation to temperature stress in natural habitats.

In rice, a transgenic approach has been adopted to improve cold stress tolerance. We update the engineering cold stress-tolerant rice since 2011 (Table 2). Based on the functions of these genes in cold stress tolerance in rice, they can be divided into positive regulatory genes and negative regulatory genes. Positive regulatory genes include *OsCAF1B* [114], *OsSHMT1* [115], *OsUGT90A1* [116], *OsPYL10* [117], *OsRBGD3* [118], *OsPYL3* [119], *OsSAPK6* [120], *OsPUB2/3* [121], *OsRAN1* [122], *OsRAN2* [123], *OsZFP182* [124], *OsCTZFP8* [125], *OsbZIP46* [120], *ONAC095* [126], *OsNAC5* [127], *OsWRKY76* [128], *OsTERF2* [129] and *OsMYB2* [130]. In addition, negative regulatory genes include *OsMYB30* [131], *OsbZIP52* [132], *OsSPX1* [129] and *OsWRKY45* [133]. Based on the four signaling pathways above, these engineered genes function downstream of ABA signaling, ROS signaling, and CBF regulon under chilling stress response in rice (Table 2). Therefore, these genes are responsible for ROS homeostasis, proton transport, pectin degradation, and trehalose biosynthesis under cold stress in rice.

**Table 2:** List of transgenic chilling stress rice lines since 2011.

Genes	Proteins	Signaling Pathways	Chilling Stress Regulation	References
*OsCAF1B*	CCR4-associated factor 1	ROS	Positive	[114]
*OsSHMT1*	Serine hydroxymethyltransferase	ROS	Positive	[115]
*OsUGT90A1*	UDP-glycosyltransferase	ROS	Positive	[116]
*OsPYL3*	pyrabactin resistance-like	ABA	Positive	[119]
*OsRBGD3*	RNA-binding glycine-rich protein	ABA	Positive	[118]
*OsPYL10*	ABA receptor 10	ABA	Positive	[117]
*OsSAPK6*	SNF-1 related protein kinase 2	ABA	Positive	[120]
*OsPUB2/3*	U-box E3 Ub ligases	CBF	Positive	[121]
*OsRAN1*	small GTPase	ROS	Positive	[122]
*OsRAN2*	small GTPase	ROS	Positive	[123]
*OsZFP182*	TFIIIA-type zinc finger protein	ROS	Positive	[124]
*OsCTZFP8*	C2H2 zinc finger protein	ROS	Positive	[125]
*OsbZIP46*	bZIP transcription factor	ABA	Positive	[120]
*OsbZIP52*	bZIP transcription factor	ABA	Negative	[132]
*ONAC095*	NAC transcription factor	ABA	Positive	[126]
*OsNAP*	NAC transcription factor	ABA	Positive	[134]
*OsNAC5*	NAC transcript factor	ROS	Positive	[127]
*OsMBY30*	MYB transcription factor	ROS	Negative	[131]
*OsMYB2*	MYB transcription factor	ROS	Positive	[130]
*OsWRKY76*	WRKY transcript factor	ROS	Positive	[128]
*OsWRKY45*	WRKY transcript factor	ROS	Negative	[133]
*OsTERF2*	ethylene response factor	ROS	Positive	[88]
*OsSPX1*	SPX domain protein	ROS	Positive	[129]
*OsBURP16*	Polygalacturonase 1β	ROS	Negative	[135]
*OsAOX1a*	alternative oxidase 1	ROS	Positive	[136]
*OsAPX2*	ascorbate peroxidase	ROS	Positive	[137]
*OsAPXa*	ascorbate peroxidase	ROS	Positive	[76]
*OVP1*	V-PPase	ROS	Positive	[138]
*OsTPS1*	trehalose-6-phosphate synthase	CBF	Positive	[139]

## 7. Conclusions and Perspectives

This review analyzes the convergence in cold sensors PM-bound calcium channels and receptor-like protein kinases in cold stress sensing in *Arabidopsis* and rice. Identifying these proteins in calcium signaling and MAPK cascades will be helpful to strengthen the cold-induced perception mechanisms in *Arabidopsis* and rice. On the other hand, there is convergence in calcium signaling, MAPK cascade signaling, ICE-CBF transcriptional pathways, and divergence in phospholipid signaling and ROS signaling. These signaling pathways have evolved into distinctive and integrative networks in cold stress responses in both species.

In the future, regulon engineering will be a novel strategy by using a master regulatory switch to cause transcriptional changes in the cold response of rice [140]. An efficient engineering approach will be to generate constitutively active mutant genes, for example, by deleting the inhibitory domains of transcription factors or changing phosphorylation mimicking/depriving status in the signal transducers such as MAPK, MAPKK, or receptor kinases. Meanwhile, to prevent constitutive overexpression of cold-responsive genes from consuming extra energy and producing undesirable traits, tissue-specific and cold stress-inducible promoters will be beneficial for the genetic improvement of cold tolerance in rice. Finally, a multi-genes site-specific assembly system will be developed. Elite constructs with multiple stress tolerant genes via CRISPR-Cas9 strategy should be designed to enhance rice crop productivity to cold stress.

## Figures and Tables

**Table 1:** List of transgenic freezing stress *Arabidopsis* lines since 2011.

Genes	Proteins	Signaling Pathways	Freezing Stress Regulation	References
*AtLCBK1*	long-chain base kinases	Phospholipid	Positive	[102]
*AtBZR1*	brassinazole-resistant 1	CBF	Positive	[103]
*AtCRF4*	cytokinin response factor 4	CBF	Positive	[104]
*AtOST1*	open stomata 1	CBF	Positive	[85]
*AtACBP6*	Acyl-coenzyme A-binding protein 6	Phospholipid	Positive	[107]
*AHP2/3/5*	histidine phosphotransfer protein 2/3/5	CBF	Positive	[28]
*ARR22*	*Arabidopsis* response regulator	CBF	Positive	[105]
*AtTCF1*	Tolerant to Chilling and Freezing	ROS	Negative	[106]
*AtVOZ2*	vascular one zinc-finger protein	ROS	Negative	[108]
*AtCCA1*	circadian clock-associated 1	CBF	Positive	[109]
*AtMYB96*	MYB transcription factor	CBF	Positive	[110]
*AtFTL1*	AP2 transcription factor	CBF	Positive	[111]
*AtPMEI13*	pectin methyl-esterase inhibitor	ROS	Negative	[112]
*AtSAG101*	lipase-like regulators	Phospholipid	Negative	[113]
*AtEDS1*	lipase-like regulators	Phospholipid	Negative	[113]
*AtPAD4*	lipase-like regulators	Phospholipid	Negative	[113]

## Data Availability

Not applicable.
